# Chewing efficiency, nutritional status, and frailty in geriatric patients: a cross-sectional study

**DOI:** 10.1186/s12877-026-07668-4

**Published:** 2026-05-23

**Authors:** Wiebke Könning, Birte Holtfreter, Stefanie Samietz, Thomas Kocher, Maximilian König

**Affiliations:** 1 Department of Internal Medicine D-Geriatrics, University Medicine Greifswald, Walther-Rathenau-Str. 49, Greifswald, 17475 Germany; 2 Department of Restorative Dentistry, Periodontology and Endodontology, University Medicine Greifswald, Greifswald, Germany; 3 Department of Prosthetic Dentistry, Gerodontology and Biomaterials, University Medicine Greifswald, Greifswald, Germany

**Keywords:** Dentition, Prosthetic status, Frailty, Nutritional status, Mastication, Gerodontology

## Abstract

**Background:**

Chewing function is critical for maintaining health and well-being in older adults. Impaired chewing efficiency has been linked to systemic conditions such as stroke, respiratory disease, and cognitive decline, as well as increased frailty and its progression. Tooth loss and partial dentition often lead older adults to avoid hard-to-chew, fibre-rich foods, thereby increasing their risk of malnutrition. Despite its importance, chewing function remains under-recognized in geriatric assessments. This study aimed to evaluate chewing efficiency and its association with frailty and nutritional status in geriatric patients.

**Methods:**

Cross-sectional study of 150 consecutive patients at an acute geriatric clinic in Germany. Participants underwent comprehensive geriatric and dental examinations, including a frailty-assessment using the Clinical Frailty Scale (CFS) and a nutritional screening, the Mini Nutritional Assessment short form (MNA). Chewing efficiency was measured using a two-color chewing gum mixing test.

**Results:**

Among the 150 patients (mean age 82.3 ± 7.6 years, 63.3% female), 28.7% were edentulous. Malnutrition was present in 44.6% of patients, and 47.5% were at risk of malnutrition. Frailty was identified in 81.2% of patients. Lower chewing efficiency was significantly associated with higher frailty (B = 1.36; 95% CI: 0.30, 2.42) and poorer nutritional status (B=-2.46; 95% CI: -4.78, -0.14).

**Conclusion:**

Chewing efficiency, primarily an indicator of oral health, also closely reflects systemic frailty. Preserving oral function and chewing ability may be important for maintaining overall health in older adults. Given the high prevalence of oral health deficits in frail older adults, incorporating a brief dental assessment into routine geriatric care is both feasible and warranted.

**Trial registration:**

The study received approvel from the Ethics Committee of the Universitätsmedizin Greifswald, and was registered in the German Clinical Trials Register (DRKS) on 17th April 2024 (registration number: DRKS00033778).

**Supplementary Information:**

The online version contains supplementary material available at 10.1186/s12877-026-07668-4.

## Introduction

Due to preventive dental care, particularly in the management of caries and periodontitis, current and future generations of older adults are retaining their natural teeth longer than previous generations [1]. Consequently, oral health in old age - especially among care-dependent individuals - is gaining importance [2]. This development is reinforced by demographic change - the proportion of people aged 80 and older is steadily increasing and is projected to soon exceed 10% [3] - which is expected to place geriatric patients at the centre of dental care [2, 4].

Chewing function plays a crucial role in overall well-being and healthy ageing [5]. Not only nutritional status is closely related to chewing function [6]. There is evidence linking impaired chewing function to a range of systemic conditions, including stroke, chronic pulmonary disease, carotid atherosclerosis, and depression [7]. Furthermore, reduced chewing capacity may contribute to the development of cognitive decline and neurodegenerative disorders, including dementia [8–10]. Previous studies also indicated that reduced chewing efficiency is associated with an elevated risk of frailty and its progression [11–13], a state of diminished physiological reserve and resilience to stressors due to age-related multisystem decline [14]. As a systemic, age-related phenotype [15], frailty inherently affects the oral cavity. Although the relationship between oral health and frailty has recently gained some attention in translational dental research [16], and concepts such as dental functional capacity and the oral frailty phenotype have been proposed [17, 18], frailty remains largely overlooked in routine dental practice. On the other hand, in medical and geriatric diagnostics, including the comprehensive geriatric assessment (CGA) [9], despite increasing evidence and recognition of the importance of oral health for systemic well-being, structured assessments of oral and chewing function remain neglected.

Chewing efficiency is a complex integrative function, influenced by numerous interdependent factors such as dentition (number, morphology, and health), occlusion, muscular strength and coordination, salivation, temporo-mandibular joint function, and neuromuscular control. Multiple subsystems interact dynamically to ensure effective food processing. Accordingly, chewing inefficiency can be regarded as a complex, multifactorial condition, closely associated with geriatric syndromes - particularly frailty [16]. Furthermore, chewing function is closely related to nutritional status [6]. Edentulous or partially dentate individuals often avoid fibre-rich foods that are difficult to chew, increasing their risk to develop nutrient deficiencies [19, 20]. For example, a longitudinal study from Japan showed that declining oral function - particularly chewing performance - contributed to malnutrition and weight loss in older adults [21]. Likewise, reduced chewing function has been shown to be associated with food avoidance and digestive disorders in older adults [22, 23]. Indeed, there is preliminary evidence, often from studies with small sample sizes, that preserving chewing function is not only a preventive measure, but also a modifiable factor that influences functional decline and the onset of frailty in old age [24–27]. Against this background, this study aimed to investigate associations between chewing efficiency, frailty, and nutritional status among 150 consecutive geriatric patients.

## Methods

### Study sample

The study sample comprised 150 consecutive patients admitted to an acute geriatrics hospital in Germany between February and May 2024 (Supplementary Fig. 1). The sample size was determined based on feasibility considerations and in line with comparable studies, which typically included fewer than 150 participants. We sought to include patients broadly, so that the sample would reflect the geriatric patient population typically seen in this setting. The only inclusion criteria were age ≥ 65 years and admission to the acute geriatrics department. Exclusion criteria included severe cognitive impairment or other conditions preventing informed consent or compliance (e.g., advanced dementia, delirium), palliative end-of-life care, haemophilia, or carrying a cardiac implant card (relevant for periodontal assessment). All participants or their legal representatives provided written, informed consent.

Based on standardised protocols, a structured interview, a comprehensive geriatric assessment, and a full-mouth dental examination were conducted.

### Dental and periodontal examination

The dental examination was implemented on the ward in the patients’ rooms, either in bed or seated (including in a wheelchair, if applicable), according to their preferences. Dental examinations were closely aligned with the methodology of dental examination in the Study of Health in Pomerania (SHIP-TREND) [28]. Dental examinations were performed using a magnifying glass (2-fold magnification) and light, two dental mirrors, and a manual periodontal probe (PCPUNC15, Hu-Friedy, USA).

The tooth status was recorded for all teeth except third molars. The number of teeth and the number of opposing pairs, including natural teeth, bridges and dental implants but excluding third molars were calculated.

Probing depths (PD), clinical attachment levels (CAL), and bleeding on probing were recorded at six sites per tooth (full-mouth). Measurements were rounded to the next whole millimeter. PD was measured as the distance between free gingival margin (FGM) and pocket base. If the cemento–enamel junction (CEJ) was located sub-gingivally, CAL was calculated as PD minus the distance between FGM and CEJ. If recession was present at the examined site, CAL was directly measured as the distance between CEJ and the pocket base. Where the determination of the CEJ was indistinct (wedge-shaped defects, fillings, and crown margins), CAL was not recorded. At the patient level, mean PD, mean CAL and the proportion of sites with bleeding on probing were calculated. The Centers for Disease Control and Prevention (CDC)/American Academy of Periodontology (AAP) classification was determined [29] and patients were categorised as having no or mild, moderate, or, severe periodontitis.

Coronal caries was diagnosed visually with gentle probing at the surface level (occlusal, distal, buccal, mesial, palatinal/lingual; excluding third molars) The number of decayed (only dentine caries), filled (excluding crowned front teeth after trauma) or missing teeth (excluding persisting teeth of the first dentition, teeth missing due to trauma or orthodontics, missing teeth due to non-eruption) was determined (DMF-T score). Self-reported dry mouth (yes/no) was obtained via interview.

All dental assessments were conducted by a single licensed dentist who had undergone training and calibrations against the gold standard examiner of SHIP-TREND. For the assessment of tooth and prosthetic status, both inter- and intra-rater agreement were excellent (κ = 1.0). The intra-rater and inter-rater agreement for both CAL and PD measurements were 0.96 and 0.97, respectively. For coronal caries examinations, Cohen’s κ was 0.97 (intra-examiner) and 0.98 (inter-examiner).

### Chewing efficiency

**Fig. 1:**
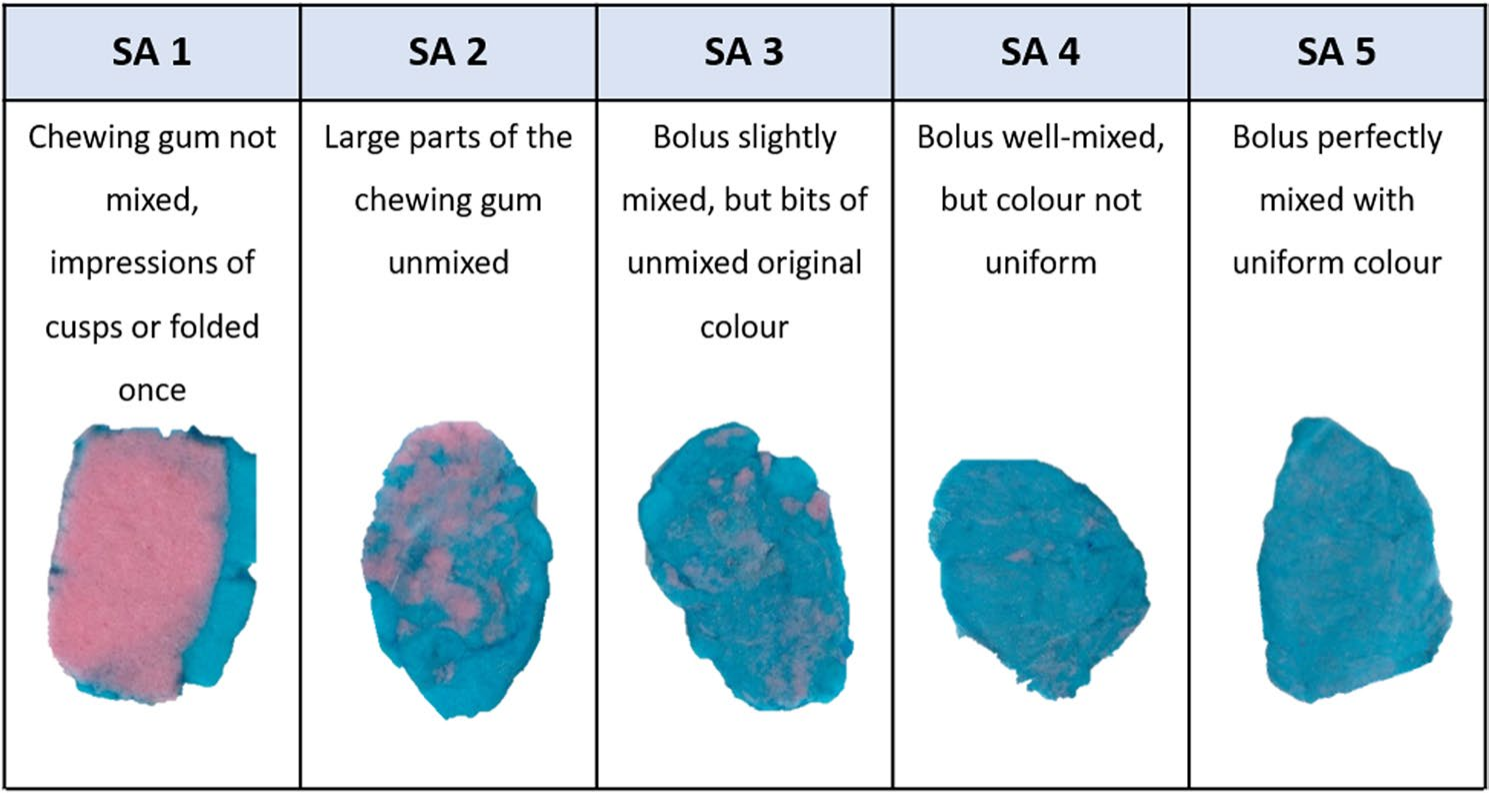
Subjective analysis (SA) of chewing gums. The images illustrate representative examples for each category. None of the patients in this study reached category SA 5

Chewing efficiency was assessed using a two-colour chewing gum mixing test (Hue-check Gum©). This chewing gum was developed and produced for Orophys GmbH (Muri b, Bern, Switzerland). Patients were instructed to chew 2 gums (pink and blue) for 20 chewing cycles. The bolus was then dried, placed in a transparent plastic bag, and flattened to a thickness of 1 mm. The gums were evaluated using the Subjective Analysis (SA) Score; see Fig. 1 [30]. Intra-rater reliability was assessed using a weighted kappa, yielding κ = 0.924 (95% CI: 0.616–1.000) between two measurement occasions spaced over one month.

Both sides of the flattened chewed samples were scanned using a flatbed scanner and analysed with the ViewGum© software (Version 4.1.2.1) to determine the “Variance of Hue” (VOH). A higher VOH indicates lower colour mixing and thus reduced chewing efficiency. The VOH value was categorised as acceptable (0.0-0.45), poor (> 0.45–0.75), and very poor (> 0.75-1.0).

### Comprehensive geriatric assessment

The comprehensive geriatric assessment included a nutritional screening using the Mini Nutritional Assessment short form (MNA; categorised as 0–7 points [malnutrition], 8–11 points [risk for malnutrition], 12–14 points [normal] [31]), the Geriatric Depression Scale (GDS); categorised as 0–5 points [normal]; 6–10 points [mild to moderate depression]; 11–15 points [severe depression] [32], and a cognitive and delirium screening test (4AT Score; categorised as ≥ 4 points [possible delirium]; 1–3 points [possible cognitive impairment]; 0 points [normal] [33]). The patient’s level of independence in basic activities of daily living, such as feeding, bathing, mobility, and toileting was assessed using the Barthel Index (categorised as 0–30 points [largely dependent on care], 35–80 points [in need of help], 85–95 points [selectively in need of help]) [34].

### Clinical frailty scale

The Clinical Frailty Scale (CFS) was used to assess frailty [35]. It can be applied by any adequately trained healthcare professional, with patients rated on a scale from 1 (very fit) to 9 (terminally ill).The assessment is not based on the patient’s condition during the acute phase of illness, but rather on their functional ability as reported over the two weeks preceding admission [36]. The distribution of the CFS can be seen in Supplementary Fig. 2. Given the predominantly frail patient population, categorisation was performed into the following three groups: CFS 1– 4: robust to pre-frail, CFS 5–6: moderate frailty, with mild to moderate limitations in independence, and CFS 7– 8: severely frail, with substantial limitations in independence, requiring comprehensive care support.

### Covariates

Additional clinical data were extracted from patients’ medical records, including the main diagnosis (categorised as cardiovascular diseases, delir/cognitive deficits, fractures/total endoprosthesis, pulmonary diseases, diseases of the internal organs, depression, stroke, others), and the number of diseases (including chronic lung disease, asthma, kidney disease, cancer (excluding low-grade skin cancer), joint disease (all forms, degenerative and inflammatory, such as osteoarthritis and rheumatism), high blood pressure, diabetes, heart attack, heart insufficiency; categorised as 0–4 vs. 5–11).

Education was recorded as < 10, 10, or > 10 years of schooling. Marital status was categorised as follow: married/registered civil partnership; married/registered civil partnership, but separated from partner; divorced/registered civil partnership annulled; widowed/registered civil partner deceased; in a committed relationship; or single. For the regression models, marital status was converted to reflect whether or not the patient was living with a partner or alone. The living situation prior to hospital admission was categorised as ‘at home’, ‘in a retirement/nursing home’ or ‘in an assisted living facility’. Assignment of care level was recorded as ‘no’, ‘yes’ or ‘don’t know’. Professional home care service was recorded as ‘no’, ‘yes, outpatient care service’ or ‘yes, day care’. Smoking status was defined as never versus former or current smoking. The Body Mass Index (in kg/m^2^) was calculated based on information from the patient’s medical record.

### Statistical analysis

Data were reported as mean ± standard deviation (SD), median (25%; 75 percentiles), or as numbers (percentages). Group differences were assessed using Chi squared tests and Kruskal-Wallis tests.

Firstly, we investigated the relationship between chewing efficiency (measured by VOH) and frailty (measured by CFS) using linear regression analyses. Two models were constructed: the crude model included only VOH. For the fully adjusted model, selection of covariates was guided by directed acyclic graphs (DAGs) (Supplementary Fig. 3). Accordingly, the minimal sufficient adjustment set included age (continuous), sex, school education (categorical), marital status (categorical), body mass index (continuous), number of diseases (categorical), 4AT score (categorical), and Geriatric Depression Scale score (categorical). Secondly, we evaluated the association between chewing efficiency (measured by VOH) and nutritional status (measured by the continuous MNA score). Selection of covariates was again informed by a DAG (Supplementary Fig. 4): the minimal sufficient adjustment set included age (continuous), sex, school education, marital status, body mass index (continuous), number of diseases (categorical), 4AT score (categorical), and Geriatric Depression Scale score (categorical). VOH, the primary exposure variable, was modelled both as a continuous variable (Table 3) and as a categorical variable (acceptable, poor, very poor; Supplementary Table 3). Thirdly, we estimated the association between the nutritional status (measured by the continuous MNA score) and frailty (measured by the continuous CFS), adjusting for age (continuous), sex, school education, marital status, body mass index (continuous), VOH (continuous), number of diseases (categorical), 4AT score (categorical), and Geriatric Depression Scale score (categorical) (for the DAG refer to Supplementary Fig. 5). For all models, non-linear forms of continuous variables were checked using the Bayesian information criterion, post hoc ANOVA tests and graphical analysis, and were found to be absent. For all models, we reported beta coefficients with 95% confidence intervals (CI) and *p*-values.

All statistical analyses were performed using Stata/SE (StataCorp LLC, College Station, TX, USA, Version 17.0) and R version 4.5.1. The significance level (α) was set at 5%.

## Results

### Patient characteristics

The study cohort comprised 150 patients with a mean age of 82.4 years (SD 7.6; range 65–100 years); 65.3% were female (Table 1). Based on the CFS, 18.8% of participants were classified as robust to pre-frail, 49.0% as moderately frail, and 32.2% as severely frail (Supplementary Fig. 2). The average Barthel Index score was 51.7, indicating moderate to severe impairment in basic activities of daily living. Prior to hospital admission, the majority of patients (84.7%) had been living at home. A formal care level had been assigned in 59.7% of cases, and 39.5% received regular assistance from professional home care services. There was a wide variety of primary treatment diagnoses.

**Table 1:** Patient characteristics: overall (*n* = 150) and stratified by chewing efficiency, as measured by the Variance of Hue (VOH; *n* = 122)

			Chewing efficiency according to Variance of Hue (VOH)			
	*N*	Total	Acceptable (VOH 0.0-0.45) (*n* = 35)	Poor (VOH > 0.45–0.75) (*n* = 43)	Very poor (VOH > 0.75-1.0) (*n* = 44)	*p*-value
Age, years	150	82.4 ± 7.6	79.6 ± 7.7	83.7 ± 7.7	83.1 ± 7.2	0.034
Female sex	150	98 (65.3%)	22 (62.9%)	32 (74.4%)	26 (59.1%)	0.298
School education	146					
< 10 years		82 (56.2%)	16 (45.7%)	27 (62.8%)	25 (58.1%)	
10 years		41 (28.1%)	13 (37.1%)	12 (27.9%)	9 (20.9%)	
> 10 years		23 (15.8%)	6 (17.1%)	4 (9.3%)	9 (20.9%)	0.312
Marital status	142					
Married		54 (38.0%)	15 (44.1%)	18 (42.9%)	13 (33.3%)	
Married, but separated from partner		1 (0.7%)	0 (0%)	0 (0%)	1 (2.6%)	
Divorced		11 (7.8%)	5 (14.7%)	2 (4.8%)	2 (5.1%)	
Widowed		59 (41.6%)	11 (32.4%)	18 (42.9%)	17 (43.6%)	
In a relationship		8 (5.6%)	3 (8.8%)	2 (4.8%)	1 (2.6%)	
Single		9 (6.3%)	0 (0%)	2 (4.8%)	5 (12.8%)	0.239
Living situation	150					
at home		127 (84.7%)	30 (85.7%)	37 (86.1%)	38 (86.4%)	
retirement/nursing home		5 (3.3%)	2 (5.7%)	1 (2.3%)	1 (2.3%)	
assisted living facility		18 (12.0%)	3 (8.6%)	5 (11.6%)	5 (11.4%)	0.896
Care level assigned	149					
No		50 (33.6%)	15 (42.9%)	13 (30.2%)	12 (27.9%)	
Yes		89 (59.7%)	18 (51.4%)	28 (65.1%)	27 (62.8%)	
Don’t know		10 (6.7%)	2 (5.7%)	2 (4.7%)	4 (9.3%)	0.576
Professional home care services	119					
No		66 (55.5%)	20 (69.0%)	15 (41.7%)	18 (54.6%)	
Yes, outpatient care service		47 (39.5%)	5 (17.2%)	19 (52.8%)	15 (45.4%)	
Yes, day care		6 (5.0%)	4 (13.8%)	2 (5.6%)	0 (0%)	0.014
Smoking status	150					
Never		100 (66.7%)	18 (51.4%)	32 (74.4%)	32 (72.7%)	
Former or current		50 (33.3%)	17 (48.6%)	11 (25.6%)	12 (27.3%)	0.062
Body mass index, kg/m^2^	148	27.2 ± 6.5	28.7 ± 8.3	26.7 ± 5.0	26.7 ± 6.4	0.567
Number of diseases *	150					
0–4		129 (86.0%)	32 (91.4%)	39 (90.7%)	37 (84.1%)	
5–11		21 (14.0%)	3 (8.6%)	4 (9.3%)	7 (15.9%)	0.511
4AT Score	146					
≥ 4 points (possible delirium)		26 (17.8%)	3 (8.8%)	6 (14.0%)	9 (22.0%)	
1–3 points (possible cognitive impairment)		60 (41.1%)	11 (32.4%)	16 (37.2%)	21 (51.2%)	
0 points (normal)		60 (41.1%)	20 (58.8%)	21 (48.8%)	11 (26.8%)	0.070
Barthel index (BI)	150	51.7 ± 17.6	57.1 ± 17.8	54.2 ± 17.9	50.7 ± 16.3	0.236
0–30 points	150	22 (14.7%)	2 (5.7%)	6 (14.0%)	7 (15.9%)	
35–80 points		124 (82.7%)	30 (85.7%)	37 (86.0%)	36 (81.8%)	
85–95 points		4 (2.7%)	3 (8.6%)	0 (0%)	1 (2.3%)	0.172
Geriatric Depression Scale (GDS)	124					
0–5 points (normal)		84 (67.7%)	22 (71.0%)	30 (75.0%)	17 (53.1%)	
6–10 points (mild to moderate depression)		33 (26.6%)	6 (19.3%)	9 (22.5%)	14 (43.8%)	
11–15 points (severe depression)		7 (5.7%)	3 (9.7%)	1 (2.5%)	1 (3.1%)	0.108
Clinical Frailty Scale (CFS) Score	149	5.7 ± 1.3	5.2 ± 1.3	5.6 ± 1.2	6.1 ± 1.1	0.004
Robust to pre-frail (1–4)		28 (18.8%)	11 (31.4%)	10 (23.8%)	4 (9.1%)	
Moderately frail (5–6)		73 (49.0%)	17 (48.6%)	20 (47.6%)	22 (50.0%)	
Severely frail (7–9)		48 (32.2%)	7 (20.0%)	12 (28.6%)	18 (40.9%)	0.095
Mini Nutritional Assessment (MNA) Score	138	8.1 ± 2.8	9.2 ± 2.7	8.2 ± 3.1	7.7 ± 2.4	0.040
12–14 points (normal)		11 (8.0%)	4 (12.1%)	6 (14.3%)	1 (2.6%)	
8–11 points (risk for malnutrition)		66 (47.8%)	21 (63.6%)	17 (40.5%)	19 (48.7%)	
0–7 points (malnutrition)		61 (44.2%)	8 (24.2%)	19 (45.2%)	19 (48.7%)	0.084

Data are reported as mean ± standard deviation or numbers (percentages)

*P*-values were retrieved from Chi-squared or Kruskal-Wallis tests

*Abbreviations*: *VOH* Variance of Hue, *4AT* 4 ‘A’s Test (Alertness, Abbreviated Mental Test-4, Attention, Acute change)

* including chronic lung disease, asthma, kidney disease, cancer (excluding low-grade skin cancer), joint disease (all forms, degenerative and inflammatory, such as osteoarthritis and rheumatism), high blood pressure, diabetes, heart attack, heart insufficiency

Cognitive screening using the 4AT identified possible cognitive impairment or delirium in 58.9% of patients. Nutritional status, as assessed by the MNA, was compromised in a substantial proportion of patients: 44.2% were malnourished, while 47.8% were at risk of malnutrition.

In bivariate analyses (Supplementary Table 1), frailty status was associated with several variables. Mean age increased with frailty severity, and differences across frailty categories were observed for living and care situation, 4AT score, MNA score, Barthel Index, and GDS.

### Oral health status and chewing efficiency

A total of 28.7% of patients were completely edentulous (Table 2). Among dentate individuals, mean PD was 2.01 mm (SD 0.62), mean CAL was 3.57 mm (SD 1.38), and the mean number of remaining teeth was 13.0 (SD 7.8). Removable dental prostheses were used by 80.0% of patients. All oral health parameters worsened with increasing levels of frailty (Supplementary Table 2).

**Table 2:** Dental and periodontal status and oral hygiene: overall (*n* = 150) and stratified by chewing efficiency, as measured by the Variance of Hue (VOH; *n* = 122)

			Chewing efficiency according to Variance of Hue			
	*N*	Total	Acceptable (VOH 0.0-0.45) (*n* = 35)	Poor (VOH > 0.45–0.75) (*n* = 43)	Very poor (VOH > 0.75-1.0) (*n* = 44)	*p*-value
Proportion of bleeding sites, %	75	2.1 (0; 7.3)	0.8 (0; 4.6)	3.1 (0; 4.5)	5.2 (0; 11.1)	0.290
CDC/AAP classification	71					
No or mild periodontitis		4 (5.6%)	1 (4.8%)	3 (12.0%)	0 (0%)	
Moderate periodontitis		54 (76.1%)	17 (80.95%)	18 (72.0%)	11 (64.7%)	
Severe periodontitis		13 (18.3%)	3 (14.3%)	4 (16.0%)	6 (35.3%)	0.267
Mean PD, mm	76	2.01 ± 0.62	1.84 ± 0.54	2.09 ± 0.73	2.13 ± 0.55	0.228
Mean CAL, mm	72	3.57 ± 1.38	3.10 ± 1.13	3.51 ± 1.59	4.03 ± 1.18)	0.038
DMF-T score	150	24.7 ± 3.9	23.2 ± 4.3	25.1 ± 3.5	25.8 ± 3.5	0.008
Self-reported dry mouth, yes	150	90 (60.0%)	17 (48.6%)	29 (67.4%)	25 (56.8%)	0.237
Edentulism	150	43 (28.7%)	5 (14.3%)	10 (23.3%)	19 (43.2%)	0.012
Number of teeth (in dentate participants)	150	13.0 ± 7.8	16.1 ± 7.2	11.4 ± 7.7	9.9 ± 6.6	< 0.001
Number of occluding pairs	150	0 (0; 7)	7 (0; 9)	0 (0; 4)	0 (0; 1)	< 0.001
Removable prosthesis	150					
no		30 (20.0%)	11 (31.4%)	9 (20.9%)	4 (9.1%)	
in one jaw		29 (19.3%)	10 (28.6%)	4 (9.3%)	8 (18.2%)	
in both jaws		91 (60.7%)	14 (40.0%)	30 (69.8%)	32 (72.7%)	0.012
Subjective Analysis Score	124					
SA 1		56 (47.5%)	0 (%)	17 (39.5%)	39 (92.9%)	
SA 2		35 (29.7%)	11 (34.4%)	22 (51.2%)	2 (4.8%)	
SA 3		23 (19.5%)	17 (53.1%)	4 (9.3%)	1 (2.4%)	
SA 4		4 (3.4%)	4 (12.5%)	0 (%)	0 (%)	< 0.001
Toothbrushing frequency	106					
At least twice daily		75 (70.8%)	24 (80.0%)	22 (71.0%)	18 (66.7%)	
Less than twice daily		31 (29.2%)	6 (20.0%)	9 (29.0%)	9 (33.3%)	0.510
Last dental visit	147					
Within last 6 months		68 (46.3%)	19 (55.9%)	18 (42.9%)	21 (47.7%)	
Within last 12 months		42 (28.6%)	10 (29.4%)	12 (28.6%)	12 (27.3%)	
More than 12 months ago		37 (25.1%)	5 (14.7%)	12 (28.5%)	11 (25.0%)	0.676

Data are reported as mean ± standard deviation or median (25%; 75% percentiles) or numbers (percentages)

*P*-values were retrieved from Chi-squared or Kruskal-Wallis tests

*Abbreviations*: *AAP* American Academy of Periodontology, *CAL* clinical attachment level, *CDC* Centers for Disease Control and Prevention, *DMF-T* number of decayed, filled or missing teeth, *PD* probing depth, *SA* Subjective Analysis, *VOH* Variance of Hue

Based on the visual inspection of the chewed two-colour gum, 77.2% of patients showed impaired chewing efficiency (SA Scores 1 and 2; Table 2). The opto-electronic analysis yielded a mean colour mixing VOH of 0.58 (SD 0.24). The VOH differed by frailty status, increasing from 0.44 in pre-frail patients to 0.64 in severely frail patients (Supplementary Table 2).

### Relationship between chewing efficiency and frailty

We further examined the associations between chewing efficiency (VOH) and frailty, chewing efficiency and nutritional status, and nutritional status and frailty, using both univariate and fully adjusted linear regression models informed by a directed acyclic graph (DAG) (Table 3).

**Table 3:** Results from linear regression models evaluating associations between: (a) Chewing efficiency (VOH) and Clinical Frailty Score (CFS); (b) Chewing efficiency (VOH) and Mini Nutritional Assessment (MNA) score; and (c) Mini Nutritional Assessment (MNA) score and Clinical Frailty Score (CFS)

		*N*	Crude model	Fully adjusted model
a) Chewing efficiency (VOH) on Clinical Frailty Score (CFS)				
VOH	Continuous	96	B = 1.71 (0.70; 2.72) *P* = 0.001	B = 1.36 (0.30; 2.42) *P* = 0.014
b) Chewing efficiency (VOH) on Mini Nutritional Assessment (MNA) Score				
VOH	Continuous	97	B=-2.03 (-4.27; 0.21) *P* = 0.079	B=-2.46 (-4.78; -0.14) *P* = 0.041
c) Mini Nutritional Assessment (MNA) Score on Clinical Frailty Score (CFS)				
MNA Score	Continuous	96	B=-0.12 (-0.21; -0.02) *P* = 0.017	B=-0.08 (-0.17; 0.02) *P* = 0.133

Model (a) The fully adjusted model included age (continuous), sex, school education, marital status, body mass index (continuous), number of diseases (categorical), 4AT Score (categorical), and Geriatric Depression Scale (categorical). Model (b) The fully adjusted model included age (continuous), sex, school education, marital status, body mass index (continuous), number of diseases (categorical), 4AT Score (categorical), and Geriatric Depression Scale (categorical). Model (c) The fully adjusted model included age (continuous), sex, school education, marital status, body mass index (continuous), chewing efficiency (VOH), number of diseases (categorical), 4AT Score (categorical), and Geriatric Depression Scale (categorical)

For all models, we reported beta coefficients (B) with 95% confidence intervals and *p* values from linear regression models

*Abbreviations*: *CFS* Clinical Frailty Scale, *MNA* Mini Nutritional Assessment, *N* Number, *VOH* Variance of Hue

**Fig. 2:**
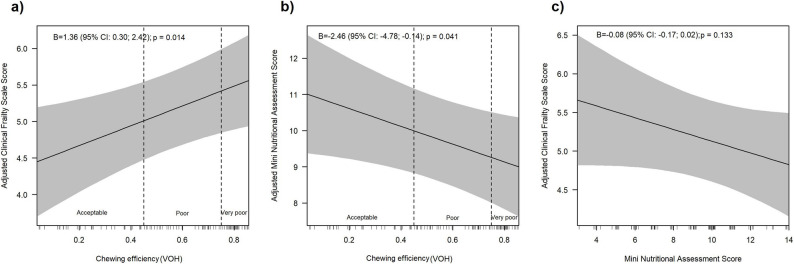
**a** Adjusted Clinical Frailty Scale Score (y-axis) by chewing efficiency as assessed by the Variance of Hue (VOH; x-axis); (**b**) Adjusted Mini Nutritional Assessment Score (y-axis) by chewing efficiency (VOH; x-axis); (**c**) Adjusted Clinical Frailty Scale Score (y-axis) by Mini Nutritional Assessment Score (x-axis). Solid lines represent predicted values and shaded areas indicate 95% confidence intervals. Higher VOH values indicate poorer colour mixing and therefore lower chewing efficiency. See Table 3 for detailed model results and adjustment sets

A significant linear relationship between chewing efficiency (VOH) and frailty (CFS) was identified (fully adjusted: B = 1.36; 95% CI: 0.30 to 2.42; Table 3a, Fig. 2a). Also, when VOH was categorised (Supplementary Table 2), a very poor chewing efficiency was associated with a higher CFS compared to acceptable chewing efficiency (B = 0.59; 95% CI: -0.09 to 1.27, *p* = 0.090).

### Relationship between chewing efficiency and nutritional status

Likewise, a significant linear association between chewing efficiency and MNA was observed (Table 3b, Fig. 2b). In the fully adjusted model, higher levels of VOH, indicating worse chewing efficiency, were significantly associated with lower MNA scores (B=-2.46; 95% CI: -4.78 to -0.14). With VOH categorized into acceptable, poor, and very poor (Supplementary Table 3), patients with very poor chewing efficiency showed significantly lower MNA scores compared to those with acceptable chewing efficiency (B=-1.36; 95% CI: -2.69 to -0.04).

### Relationship between nutritional status and frailty

In the fully adjusted model, lower MNA scores were only borderline associated with higher CFS scores. Each one-point decrease in the MNA corresponded to a 0.08-point increase in the CFS (B = -0.08; 95% CI: -0.17 to 0.02; Table 3c, Fig. 2c).

## Discussion

This study found that reduced chewing efficiency, indicated by higher VOH levels, was significantly associated with increased frailty and lower MNA scores in geriatric patients, highlighting the relevance of chewing function not only for nutritional risk but also for functional decline.

Previous research has shown that various oral health parameters, including chewing function, are significantly impaired in people with frailty [11, 12]. The present findings extend this evidence by specifically highlighting chewing efficiency as a functional marker that is consistently associated with frailty status.

Chewing efficiency represents a clinically meaningful, yet often underappreciated, functional parameter within both geriatric and dental assessment [37]. Unlike purely structural and static oral health indicators (e.g. number of teeth, occlusal pairs, score of decayed, filled, missing teeth) [16, 38, 39], it reflects the integrated performance of the masticatory system, including dentition, prosthetes, muscle strength, salivary flow, neuromotor coordination, and sensory feedback [16, 40–42].

Reduced chewing efficiency has several important implications. First and foremost, it influences nutritional intake and diet quality, as individuals with impaired chewing function tend to avoid harder, fibre-rich high-quality and protein-rich foods [37]. This dietary shift may contribute to malnutrition, weight loss, sarcopenia, and ultimately frailty [6, 23, 43]. Frailty hallmarks, such as fatigue, reduced activity, and muscle weakness - including the masticatory muscles - may further contribute to impaired chewing function [44], leading to a downward spiral of impaired nutrition, declining physical function, and increasing frailty. Consistent with this, our regression models showed significant associations between lower chewing efficiency (indicated by higher VOH values) and reduced MNA scores.

It is noteworthy that malnutrition was observed in 44.2% of patients, with an additional 47.8% being at risk of malnutrition in this cohort of typical geriatric patients [45, 46]. These observations are consistent with those reported in previous studies [47]. The causes of malnutrition in old age are multifactorial. They range from age-related declines in smell and taste perception, which can lead to a loss of appetite, to comorbidities and medication side effects. Notably, poor oral health is an important contributing factor [43]. In this context, our findings are both relevant and clinically meaningful as they confirm a close interrelationship between chewing efficiency, nutritional status, and frailty. Importantly, chewing efficiency is a modifiable factor. Interventions such as dental rehabilitation (e.g., prosthodontic treatment), dietary counselling, and masticatory training may improve oral function and, indirectly, nutritional status and physical resilience [37]. This positions chewing efficiency not only as a marker of frailty but also as a potential target for intervention within multidisciplinary geriatric care.

In clinical practice, standardised instruments for assessing chewing efficiency are rarely used, and those available are often impractical for routine use [48]. The chewing gum colour-mixing test used in our study proved easily implementable even in our predominantly frail, geriatric study population. It requires minimal time, no specialist training, and can be conducted in any healthcare setting. Furthermore, the test is suitable for denture wearers, as the material does not adhere to teeth or prostheses [49]. Based on our findings, the Hue-check Gum test appears particularly well suited for use in geriatric settings to rapidly identify dental treatment needs - especially among patients who, due to access barriers, no longer attend dental visits regularly. However, despite its promising utility, this relatively new method of assessing chewing efficiency has not yet undergone validation in larger cohorts, nor have reference values been established for different age or dentition groups to guide clinical interpretation [50].

Among the strengths of this study, the broad examination spectrum, including common geriatric tools and comprehensive oral health assessments, and the high reliability of the study examiner in dental examinations should be noted. Among the limitations, the relatively small sample size (*n* = 150) should be noted; however, this number exceeds those of many comparable investigations [12, 51, 52]. Secondly, the generalizability of the findings is restricted to geriatric patients, who may differ from the general population with regard to frailty and disease burden. However, the patient sample under consideration is representative of acute geriatrics in Germany with regard to age, sex distribution, disease spectrum, and frailty status [45, 46]. Thirdly, due to its cross-sectional nature, causality could not be established, and residual confounding factors may remain despite comprehensive adjustment. Furthermore, it was not possible to disentangle reverse causality between frailty and chewing efficiency. Longitudinal studies are needed to clarify causal directions and identify potential mediating mechanisms. However, such data are scarce in geriatric dentistry, largely due to interruptions in care continuity – especially at the transition into frailty and care dependency, which often involves relocation and disrupted dental follow-up [53]. It should also be noted that our sample included very few individuals with optimal chewing function or those at the fitter end of the frailty spectrum (CFS 1–3), limiting comparisons with healthier older adults. Nevertheless, the results of our study advance the current understanding of the relationship between oral health and frailty, and systemic health in old age.

Our results emphasize the need for an integrative approach that considers dental, nutritional, and medical-geriatric factors in combination. Within this framework, the objectively measurable functional parameter of chewing efficiency emerges as a particularly relevant clinical indicator.

The integration of chewing efficiency into frailty research aligns with a more holistic understanding of ageing, where oral health is considered an essential component of overall health rather than a separate domain [7, 54]. Future longitudinal research should investigate whether impaired chewing efficiency could be conceptualised as a distinct geriatric oral syndrome.

Frailty and chewing inefficiency appear to develop in parallel, share common risk factors, and may mutually reinforce each other. Although the direction of causality remains uncertain - whether reduced chewing function is a cause or consequence of frailty - this bidirectional relationship underscores the importance of oral health as an integral component of frailty management. A better understanding of this interaction could inform early interventions targeting both systemic and oral health deterioration. Future studies may investigate whether improvements in chewing efficiency translate into measurable benefits in frailty trajectories, functional outcomes, and quality of life.

## Conclusion

This study showed that chewing efficiency, primarily an indicator of oral health, also closely reflects systemic frailty. Preserving oral function and chewing ability may therefore be important for maintaining overall health in older adults. Given the high prevalence of oral health deficits and care needs in frail older adults, incorporating a brief dental assessment—including evaluation of chewing efficiency using Hue-Check Gums—into routine geriatric care is both feasible and warranted.

## Supplementary Information

Supplementary Material 1.

## Data Availability

Data are not publicly available due to patient confidentiality but can be requested from the corresponding author.
