# Supplementary material for: Chewing efficiency, nutritional status, and frailty in geriatric patients: a cross-sectional study
Source: BMC Geriatr. 2026 May 23;26:745. doi: 10.1186/s12877-026-07668-4 (PMC13200370; doi:10.1186/s12877-026-07668-4)
Supplement: Supplementary file 1 — Supplementary Material 1. [file 12877_2026_7668_MOESM1_ESM.docx]

# **Supplementary Material**

## Chewing Efficiency, Nutritional Status, and Frailty in Geriatric Patients: A Cross-Sectional Study

Wiebke Könning^1^, Birte Holtfreter^2^, Stefanie Samietz^3^, Thomas Kocher^2^, Maximilian König^1^

**Supplementary Figures**

**Supplementary Figure 1.** Patient recruitment flow chart.

**Supplementary Figure 2**. Histogram showing the distribution of scores on the Clinical Frailty Score (range 1 - 9); 3 = managing well, 4 = vulnerable, 5 = mildly frail, 6 = moderately frail, 7 = severely frail, 8 = very severely frail.


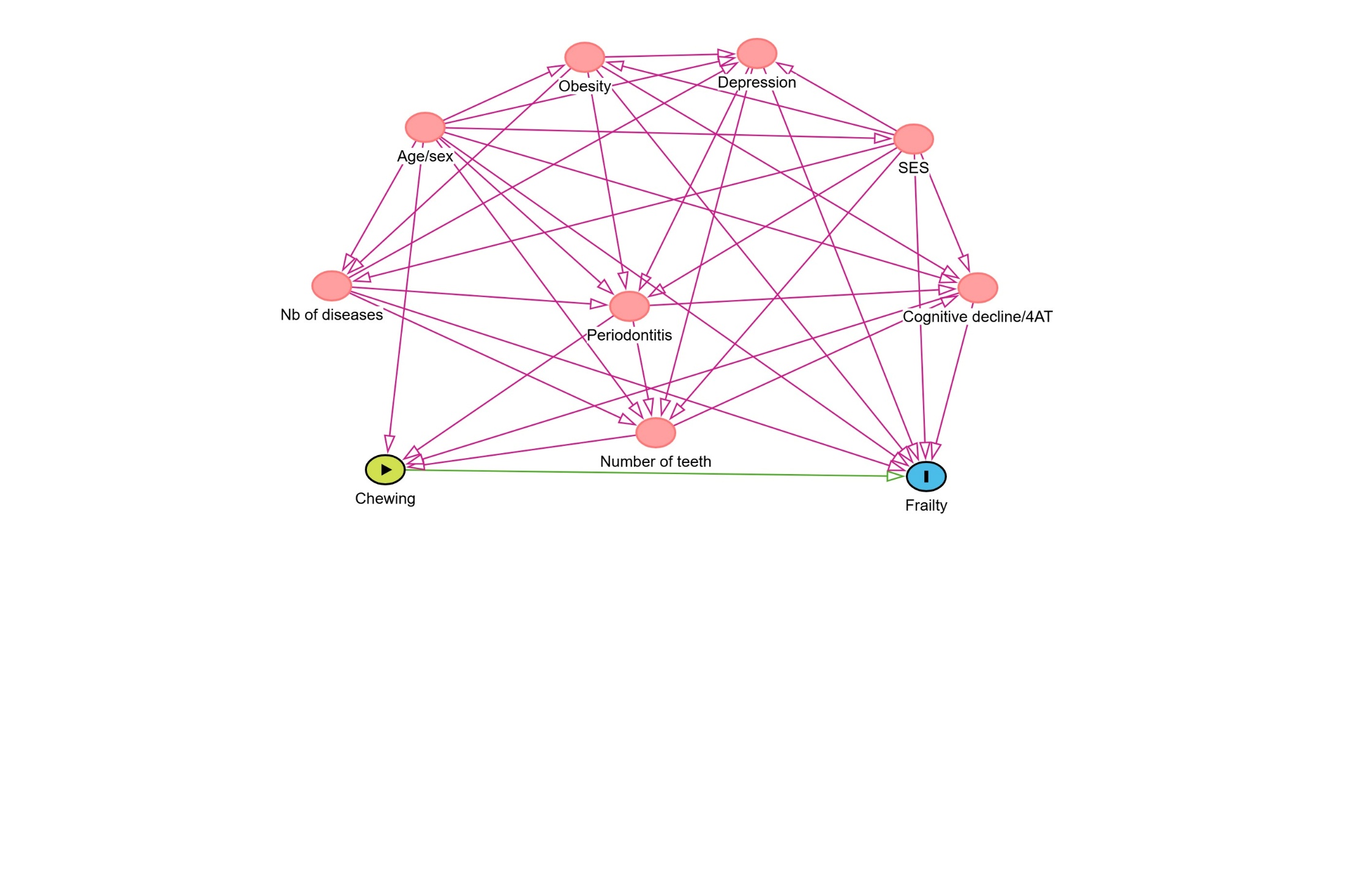


**Supplementary Figure 3.** Directed acyclic graph (DAG): Chewing efficiency on Frailty. Minimal sufficient adjustment set: age, sex, cognition (4AT Score), depression (Geriatric Depression Scale), number of diseases, obesity (body mass index), SES (school education, marital status).


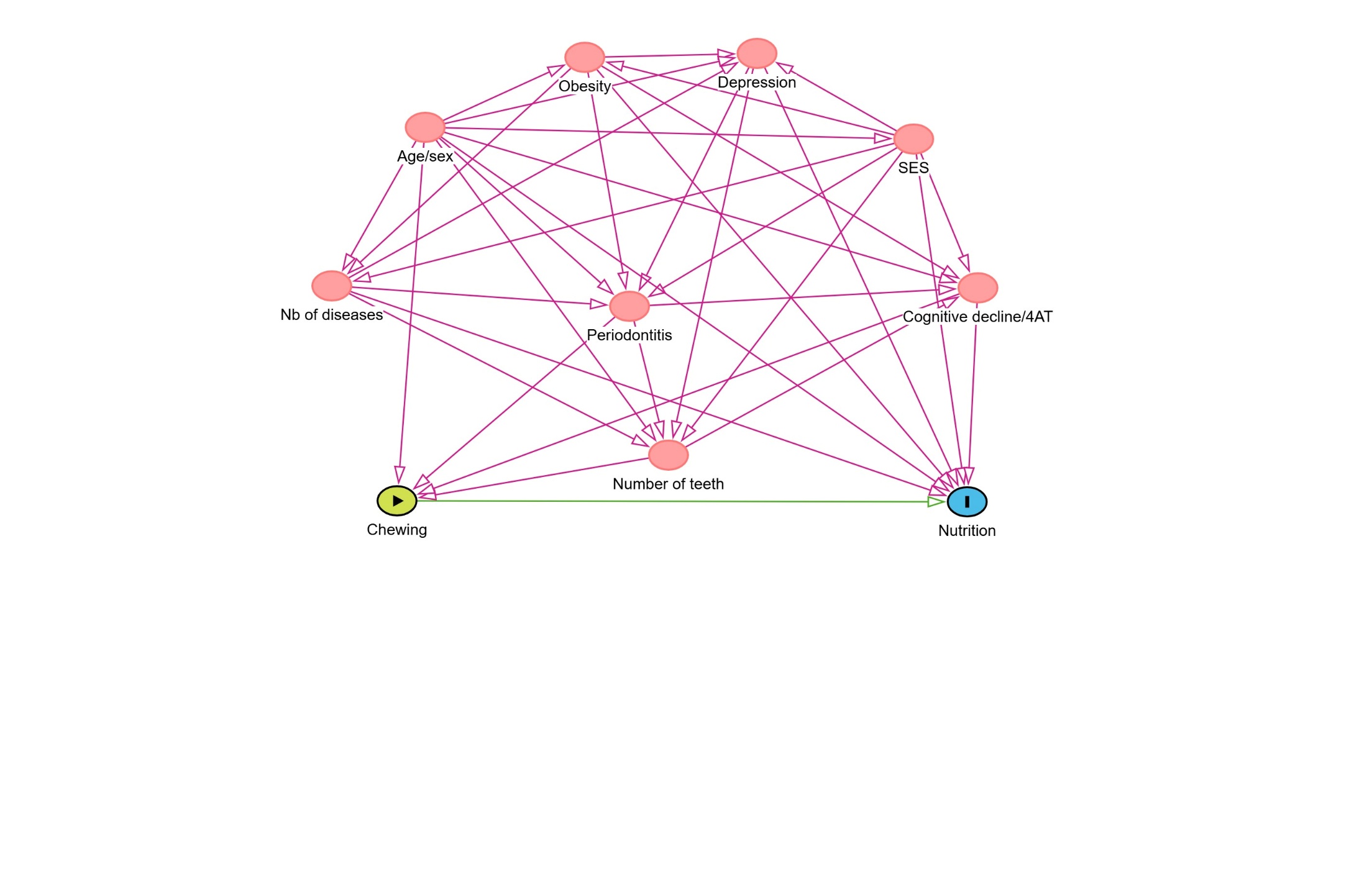


**Supplementary Figure 4.** Directed acyclic graph (DAG): Chewing efficiency on Nutrition. Minimal sufficient adjustment set: age, sex, cognition (4AT Score), depression (Geriatric Depression Scale), number of diseases, obesity (body mass index), SES (school education, marital status).


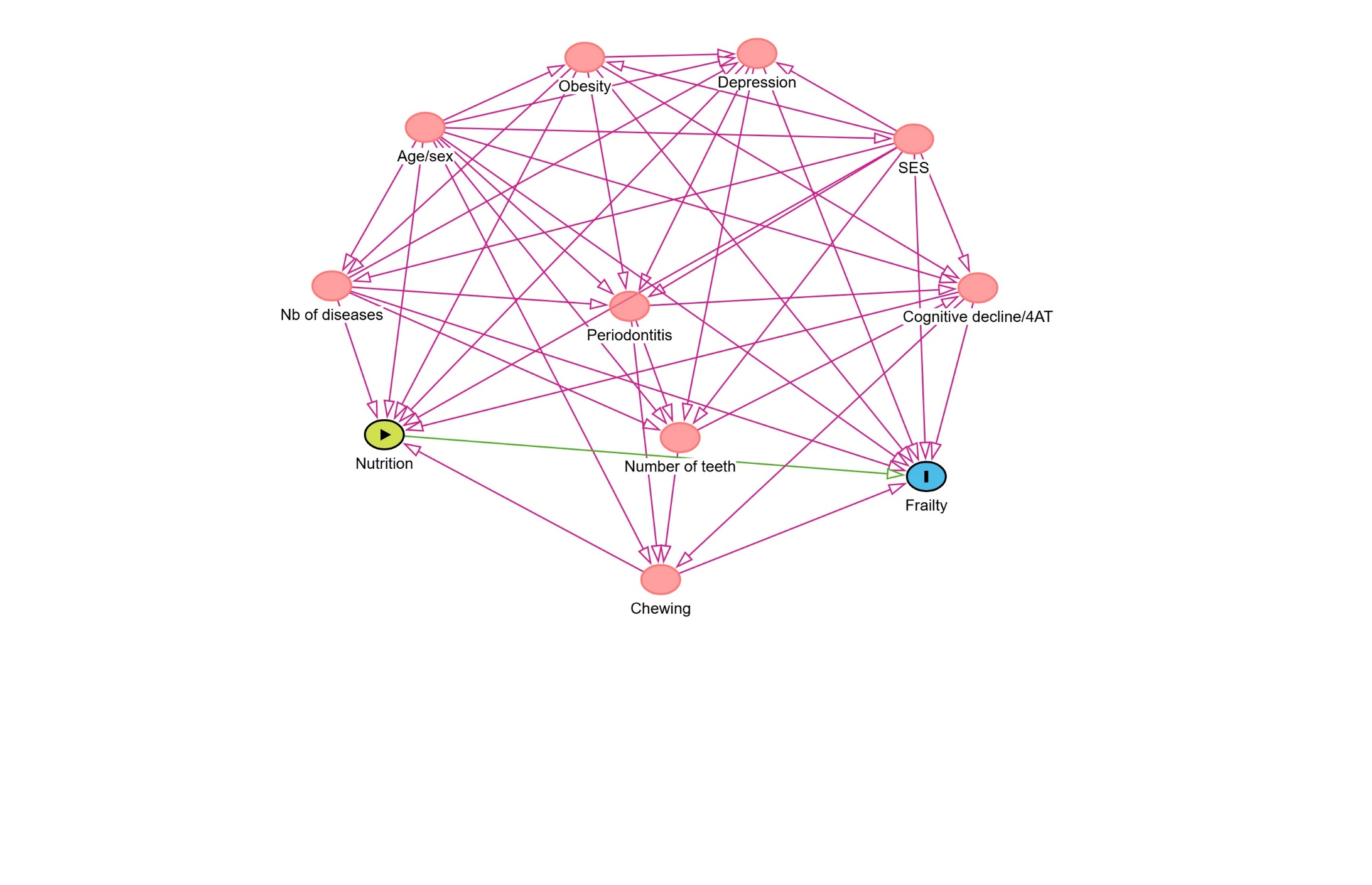


**Supplementary Figure 5.** Directed acyclic graph (DAG): Nutrition on Frailty. Minimal sufficient adjustment set: age, sex, chewing efficiency (Variance of Hue), cognition (4AT Score), depression (Geriatric Depression Scale), number of diseases, obesity (body mass index), SES (school education, marital status).

**Supplementary Tables**

**Supplementary Table 1.** Patient characteristics stratified by frailty status, as assessed by the Clinical Frailty Scale (CFS) Score (N=149).

|  | **N** | **Robust to pre-frail**  (CFS 1-4)  (N=28) | **Moderately frail**  (CFS 5-6)  (N=73) | **Severely Frail**  (CFS 7-9)  (N=48) | **p-value** |
| --- | --- | --- | --- | --- | --- |
| **Age, years** | 149 | 79.6 ± 6.9 | 81.5 ± 7.8 | 85.4 ± 6.9 | 0.002 |
| **Female sex** | 149 | 22 (78.6%) | 43 (58.9%) | 33 (68.8%) | 0.153 |
| **School education** | 145 |  |  |  |  |
| <10 years |  | 12 (44.4%) | 37 (50.7%) | 32 (71.1%) |  |
| 10 years |  | 11 (40.7%) | 21 (28.8%) | 9 (20.0%) |  |
| >10 years |  | 4 (14.8%) | 15 (20.55%) | 4 (8.9%) | 0.098 |
| **Marital status** | 141 |  |  |  |  |
| Married/registered civil partnership |  | 9 (33.3%) | 30 (44.1%) | 15 (32.6%) |  |
| Married/registered civil partnership, but separated from partner |  | 0 (0%) | 1 (1.5%) | 0 (0%) |  |
| Divorced / Registered civil partnership annulled |  | 1 (3.7%) | 6 (8.8%) | 4 (8.7%) |  |
| Widowed / Registered civil partner deceased |  | 11 (40.7%) | 23 (33.8%) | 24 (52.2%) |  |
| In a committed relationship |  | 4 (14.8%) | 4 (5.9%) | 0 (0%) |  |
| Single |  | 2 (7.4%) | 4 (5.9%) | 3 (6.5%) | 0.294 |
| **Living situation** | 150 |  |  |  |  |
| at home |  | 27 (96.4%) | 66 (90.4%) | 34 (70.8%) |  |
| retirement/nursing home |  | 0 (0%) | 0 (0%) | 4 (8.3%) |  |
| assisted living facility |  | 1 (3.6%) | 7 (9.6%) | 10 (20.8%) | 0.004 |
| **Assignment of care level** |  |  |  |  |  |
| No |  | 23 (82.1%) | 24 (32.9%) | 3 (6.4%) |  |
| Yes |  | 5 (17.9%) | 46 (63.0%) | 37 (78.7%) |  |
| Don’t know |  | 0 (0%) | 3 (4.1%) | 7 (14.9%) | <0.001 |
| **Professional home care services** | 119 |  |  |  |  |
| No |  | 19 (73.1%) | 32 (52.5%) | 15 (46.9%) |  |
| Yes, outpatient care service |  | 4 (15.4%) | 26 (42.6%) | 17 (53.1%) |  |
| Yes, day care |  | 3 (11.5%) | 3 (4.9%) | 0 (0%) | 0.024 |
| **Smoking status** | 149 |  |  |  |  |
| Never smoker |  | 18 (64.3%) | 46 (63.0%) | 35 (72.9%) |  |
| Former or current smoker |  | 10 (35.7%) | 27 (37.0%) | 13 (27.1%) | 0.510 |
| **Body mass index, kg/m^2^** | 147 | 29.4 ± 8.1 | 27.3 ± 6.1 | 26.0 ± 5.7 | 0.119 |
| **Diabetes mellitus, yes** | 147 | 10 (35.7%) | 21 (29.2%) | 14 (29.8%) | 0.807 |
| **Main diagnosis** | 146 |  |  |  |  |
| cardiovascular diseases |  | 2 (7.4%) | 9 (12.5%) | 13 (27.7%) |  |
| delir/cognitive deficits |  | 1 (3.7%) | 4 (5.6%) | 2 (4.3%) |  |
| fractures/total endoprosthesis |  | 13 (48.2%) | 21 (29.2%) | 17 (36.2%) |  |
| pulmonary diseases |  | 1 (3.7%) | 5 (6.9%) | 2 (4.3%) |  |
| diseases of the internal organs |  | 1 (3.7%) | 11 (15.3%) | 4 (8.5%) |  |
| Depression |  | 1 (3.7%) | 2 (2.8%) | 6 (12.8%) |  |
| Stroke |  | 3 (11.1%) | 7 (9.7%) | 3 (6.4%) |  |
| Others |  | 5 (18.5%) | 13 (18.1%) | 13 (27.7%) | 0.311 |
| **Number of diseases*** | 149 |  |  |  |  |
| 0-4 |  | 25 (89.3%) | 62 (84.9%) | 41 (85.4%) |  |
| 5-11 |  | 3 (10.7%) | 11 (15.1%) | 7 (14.6%) | 0.848 |
| **4AT Score** | 145 | 0.9 ± 1.5 | 1.1 ± 1.4 | 2.6 ± 3.0 | 0.001 |
| ≥4 points (possible delirium) |  | 4 (14.8%) | 7 (10.0%) | 15 (31.3%) |  |
| 1-3 points (possible cognitive impairment) |  | 5 (18.5%) | 33 (47.1%) | 21 (43.7%) |  |
| 0 points (normal) |  | 18 (66.7%) | 30 (42.9%) | 12 (25.0%) | 0.001 |
| **Barthel index** | 149 | 60.0 ± 13.2 | 57.3 ± 16.6 | 38.3 ± 13.5 | <0.001 |
| 0-30 points: largely dependent on care |  | 0 (0%) | 5 (6.9%) | 17 (35.4%) |  |
| 35-80 points: in need of help |  | 27 (96.4%) | 65 (89.0%) | 31 (64.6%) |  |
| 85-95 points: selectively in need of help |  | 1 (3.6%) | 3 (4.1%) | 0 (0%) | <0.001 |
| **Geriatric Depression Scale (GDS)** | 123 | 3.4 ± 3.2 | 4.4 ± 2.8 | 5.4 ± 3.4 | 0.034 |
| 0-5 points (normal) |  | 21 (80.8%) | 45 (69.2%) | 18 (56.2%) |  |
| 6-10 points (mild to moderate depression) |  | 4 (15.4%) | 18 (27.7%) | 11 (34.4%) |  |
| 11-15 points (severe depression) |  | 1 (3.8%) | 2 (3.1%) | 3 (9.4%) | 0.281 |
| **Mini Nutritional Assessment (MNA) Score** | 137 | 9.4 ± 2.4 | 8.3 ± 2.7 | 7.2 ± 2.7 | 0.003 |
| 12-14 points (normal) |  | 4 (14.8%) | 5 (7.5%) | 2 (4.65%) |  |
| 8-11 points (risk for malnutrition) |  | 16 (59.3%) | 32 (47.8%) | 18 (41.9%) |  |
| 0-7 points (malnutrition) |  | 7 (25.9%) | 30 (44.8%) | 23 (53.5%) | 0.189 |

***Notes*:** Data are reported as mean ± standard deviation or numbers (percentages). P-values were retrieved from Chi-squared or Kruskal-Wallis tests. *includes chronic lung disease, asthma, kidney disease, cancer (excluding low-grade skin cancer), joint disease (all forms, degenerative and inflammatory, such as osteoarthritis and rheumatism), high blood pressure, diabetes, heart attack, heart insufficiency. ***Abbreviations*:** 4AT = 4 'A's Test (Alertness, Abbreviated Mental Test-4, Attention, Acute change); CFS, Clinical Frailty Scale.

**Supplementary Table 2.** Dental and periodontal status and oral hygiene, stratified by frailty status assessed using by the Clinical Frailty Scale (CFS) Score (n=149).

|  | **N** | **Robust to pre-frail**  (CFS 1-4)  (n=28) | **Moderately frail**  (CFS 5-6)  (n=73) | **Severely Frail**  (CFS 7-9)  (n=48) | **p-value** |
| --- | --- | --- | --- | --- | --- |
| **Proportion of bleeding sites, %** | 74 | 2.6 ± 3.0 | 5.4 ± 0.2 | 7.1 ± 10.2 | 0.654 |
| **CDC/AAP classification** | 70 |  |  |  |  |
| No or mild periodontitis |  | 1 (5.3%) | 2 (6.1%) | 1 (5.6%) |  |
| Moderate periodontitis |  | 17 (89.5%) | 23 (69.7%) | 13 (72.2%) |  |
| Severe periodontitis |  | 1 (5.3%) | 8 (24.2%) | 4 (22.2%) | 0.527 |
| **Mean PD, mm** | 75 | 1.75 ± 0.35 | 2.06 ± 0.64 | 2.18 ± 0.75 | 0.154 |
| **Mean CAL, mm** | 71 | 2.91 ± 0.98 | 3.76 ± 1.28 | 3.88 ± 1.75 | 0.051 |
| **DMF-T score** | 149 | 23.1 ± 3.8  23 (20; 27) | 24.4 ± 4.1  26 (22; 28) | 26.1 ± 3.0  28 (25; 28) | 0.001 |
| **Self-reported dry mouth, yes** | 149 | 14 (50.0%) | 44 (60.3%) | 31 (64.6%) |  |
| **Edentulism, yes** | 149 | 6 (21.4%) | 19 (26.0%) | 18 (37.5%) | 0.249 |
| **Number of teeth (in dentate patients)** | 149 | 13.2 ± 9.5 | 9.8 ± 9.2 | 7.1 ± 7.7 | 0.027 |
| **Number of occluding pairs** | 149 | 5.6 ± 4.9  6.5 (0; 10) | 3.1 ± 4.3  0 (0; 6) | 2.1 ± 3.2  0 (0; 3) | 0.007 |
| **Prosthetic status** | 149 |  |  |  |  |
| no removable prosthesis |  | 9 (32.1%) | 15 (20.5%) | 6 (12.5%) |  |
| removable prosthesis in one jaw |  | 7 (25.0%) | 11 (15.1%) | 11 (22.9%) |  |
| removable prosthesis in both jaws |  | 12 (42.9%) | 47 (64.4%) | 31 (64.6%) | 0.158 |
| **Chewing efficiency (Variance of Hue)** | 121 | 0.44 ± 0.25 | 0.59 ± 0.23 | 0.64 ± 0.22 | 0.004 |
| 0.0-0.45 (acceptable) |  | 11 (44.0%) | 17 (28.8%) | 7 (18.9%) |  |
| >0.45-0.75 (poor) |  | 10 (40.0%) | 20 (33.9%) | 12 (32.4%) |  |
| >0.75-1.0 (very poor) |  | 4 (16.0%) | 22 (37.3%) | 18 (48.7%) | 0.095 |
| **Subjective Analysis Score** | 117 |  |  |  |  |
| SA 1 |  | 5 (20.8%) | 28 (49.1%) | 23 (63.9%) |  |
| SA 2 |  | 10 (41.7%) | 17 (29.8%) | 7 (19.4%) |  |
| SA 3 |  | 8 (33.3%) | 10 (17.5%) | 5 (13.9%) |  |
| SA 4 |  | 1 (4.2%) | 2 (3.5%) | 1 (2.8%) | 0.083 |
| **Toothbrushing frequency** | 105 |  |  |  |  |
| At least twice daily |  | 21 (91.3%) | 33 (62.3%) | 20 (69.0%) |  |
| Less than twice daily |  | 2 (8.7%) | 20 (37.7%) | 9 (31.0%) | 0.038 |
| **Last dental visit** | 146 |  |  |  |  |
| Within last 6 months |  | 15 (53.6%) | 38 (52.1%) | 15 (33.3%) |  |
| Within last 12 months |  | 10 (35.7%) | 15 (20.5%) | 16 (35.6%) |  |
| More than 12 months ago |  | 3 (10.7%) | 20 (27.4%) | 14 (31.1%) | 0.075 |

***Notes:*** Data are reported as mean ± standard deviation, median (25%; 75% percentiles), or numbers (percentages). P-values were retrieved from Chi-squared or Kruskal-Wallis tests. Abbreviations: AAP, American Academy of Periodontology; CAL, clinical attachment level; CDC, Centers for Disease Control and Prevention; CFS, Clinical Frailty Scale; DMF-T, number of decayed, filled or missing teeth; PD, probing depth; SA, Subjective Analysis; VOH, Variance of Hue.

**Supplementary Table 3.** Results from linear regression models evaluating associations of A) chewing efficacy (VOH; categorical) and Clinical Frailty Score (CFS); B) Chewing efficiency (VOH; categorical) on Mini Nutritional Assessment (MNA) Score; C) Mini Nutritional Assessment (MNA) Score (categorical) on Clinical Frailty Score (CFS).

|  |  | N | Crude model | Fully adjusted model |
| --- | --- | --- | --- | --- |
| 1. **Chewing efficiency (VOH) on Clinical Frailty Score (CFS)** | | | | |
| VOH | Acceptable | 30 | 0.00 (Ref.) | 0.00 (Ref.) |
|  | Poor | 38 | B=0.31 (-0.27; 0.91)  P=0.302 | B=0.25 (-0.38; 0.88)  P=0.441 |
|  | Very poor | 28 | B=0.94 (0.30; 1.58)  P=0.005 | B=0.59 (-0.09; 1.27)  P=0.090 |
| 1. **Chewing efficiency (VOH) on Mini Nutritional Assessment (MNA) Score** | | | | |
| VOH | Acceptable | 30 | 0.00 (Ref) | 0.00 (Ref) |
|  | Poor | 39 | B=-0.69 (-1.99; 0.61)  P=0.304 | B=-1.36 (-2.69; -0.04)  P=0.0468 |
|  | Very poor | 28 | B=-1.16 (-2.57; 0.24)  P=0.109 | B=-0.89 (-2.35; 0.57)  P=0.238 |
| 1. **Mini Nutritional Assessment (MNA) Score on Clinical Frailty Score (CFS)** | | | | |
| MNA Score | 12-14 points (normal) | 10 | 0.00 (Ref.) | 0.00 (Ref.) |
|  | 8-11 points (risk of malnutrition) | 52 | B=0.58 (-0.28; 1.44)  P=0.188 | B=0.20 (-0.67; 1.07)  P=0.654 |
|  | 0-7 points (malnutrition) | 34 | B=0.89 (0.0003; 1.79)  P=0.053 | B=0.38 (-0.53; 1.30)  P=0.414 |

***Notes:*** A) The fully adjusted model included age (continuous), sex, school education, marital status, body mass index (continuously), number of teeth (continuous), prosthetic status, number of diseases (categorical), 4AT Score (categorical), and Geriatric Depression Scale (categorical). B) The fully adjusted model included age (continuous), sex, school education, marital status, body mass index (continuous), number of teeth (continuous), prosthetic status, number of diseases (categorical), 4AT Score (categorical), and Geriatric Depression Scale (categorical). C) The fully adjusted model included age (continuous), sex, school education, marital status, body mass index (continuous), chewing efficiency (VOH), number of diseases (categorical), 4AT Score (categorical), and Geriatric Depression Scale (categorical). For all models, we reported beta coefficients (B) with 95% confidence intervals and p values from linear regression models. ***Abbreviations:*** CFS, Clinical Frailty Scale; MNA, Mini Nutritional Assessment; N, Number; SA, Subjective Analysis; VOH, Variance of Hue.
